# AAV5-miHTT gene therapy demonstrates suppression of mutant huntingtin aggregation and neuronal dysfunction in a rat model of Huntington’s disease

**DOI:** 10.1038/gt.2017.71

**Published:** 2017-09-14

**Authors:** J Miniarikova, V Zimmer, R Martier, C C Brouwers, C Pythoud, K Richetin, M Rey, J Lubelski, M M Evers, S J van Deventer, H Petry, N Déglon, P Konstantinova

**Affiliations:** 1 Department of Research & Development, uniQure N.V., Amsterdam, The Netherlands; 2 Department of Gastroenterology and Hepatology, Leiden University Medical Center, Leiden, The Netherlands; 3 Neurosciences Research Center (CRN), Laboratory of Cellular and Molecular Neurotherapies (LCMN), Lausanne University Hospital, Lausanne, Switzerland; 4 Department of Clinical Neurosciences, LCMN, Lausanne University Hospital, Lausanne, Switzerland

## Abstract

Huntington’s disease (HD) is a fatal progressive neurodegenerative disorder caused by a mutation in the huntingtin (*HTT*) gene. To date, there is no treatment to halt or reverse the course of HD. Lowering of either total or only the mutant HTT expression is expected to have therapeutic benefit. This can be achieved by engineered micro (mi)RNAs targeting HTT transcripts and delivered by an adeno-associated viral (AAV) vector. We have previously showed a miHTT construct to induce total HTT knock-down in Hu128/21 HD mice, while miSNP50T and miSNP67T constructs induced allele-selective HTT knock-down *in vitro*. In the current preclinical study, the mechanistic efficacy and gene specificity of these selected constructs delivered by an AAV serotype 5 (AAV5) vector was addressed using an acute HD rat model. Our data demonstrated suppression of mutant HTT messenger RNA, which almost completely prevented mutant HTT aggregate formation, and ultimately resulted in suppression of DARPP-32-associated neuronal dysfunction. The AAV5-miHTT construct was found to be the most efficient, although AAV5-miSNP50T demonstrated the anticipated mutant HTT allele selectivity and no passenger strand expression. Ultimately, AAV5-delivered-miRNA-mediated HTT lowering did not cause activation of microglia or astrocytes suggesting no immune response to the AAV5 vector or therapeutic precursor sequences. These preclinical results suggest that using gene therapy to knock-down HTT may provide important therapeutic benefit for HD patients and raised no safety concerns, which supports our ongoing efforts for the development of an RNA interference-based gene therapy product for HD.

## Introduction

Huntington’s disease (HD) is a fatal, currently untreatable, neurodegenerative disorder with a prevalence rate of 1–10 in 100 000 individuals worldwide.^1^ The presence of 40 and more CAG triplets in exon 1 of the huntingtin (*HTT*) gene has been identified as a fully penetrant trigger for the neuropathological process, which usually stretches over decades.^2, 3^ The resultant neuronal death affects primarily GABAergic medium spiny neurons in the early stage of HD, as well as neurons in other brain regions in the later stages.^4, 5^ The CAG expansion is translated into a polyglutamine (polyQ) tract in the N-terminus of the HTT protein, causing the mutant HTT to misfold and aggregate.^6^ Growing evidence suggests that the mutant HTT disturbs multiple critical cellular pathways and that its aggregation is a prerequisite to neurodegeneration. Therefore, clearance of mutant HTT is currently accepted as being key for HD treatment.^7^ Notably, Yamamoto *et al.*^8^ showed that conditional blockage of HTT expression after symptom onset results in clearance of HTT aggregates and behavioral improvements, suggesting that HD may be partially reversible.

In contrast to other progressive neurodegenerative disorders, such as Alzheimer’s or Parkinson’s disease, the monogenic nature of HD allows for the development of disease-modifying therapies that aim to halt or suppress production of the aberrant HTT. The discovery of RNA interference (RNAi) in 1998 revolutionized the progress of therapeutic interventions focusing on gene silencing at the post-transcriptional level.^9^ Since then, several groups, including ours, applied RNAi principles to design artificial small interfering (si)RNAs, short hairpin (sh)RNAs or micro (mi)RNAs that bind to the HTT transcript and reduce its translation.^10, 11^ To circumvent several challenges regarding the delivery and stable expression of RNAi products in the central nervous system, many studies have used viral vectors as delivery vehicles, which can be injected directly into the brain. reviewed in Kantor *et al.,*^12^ Adeno-associated viral (AAV) vectors are the most common vehicles of choice and a large number of AAV capsid serotypes provide cell- and tissue- specific tropism. reviewed in Srivastava^13^ For the central nervous system, studies in rodents and non human primates have shown AAV serotype 5 (AAV5) to be strong and effective in the brain, making it an attractive candidate for the RNAi-based gene transfer.^14, 15, 16, 17^ Importantly, the AAV-delivered-miRNA-based gene therapy approach comprises continual expression of artificial miRNAs following a single administration of an AAV vector, resulting in long-term HTT lowering.

To develop a disease-modifying gene therapy for HD, we have previously designed several therapeutic miRNAs targeting either both ‘total’ or preferentially the mutant HTT transcripts ‘allele-selective’ and their HTT knock-down efficiency was addressed *in vitro* and in the humanized transgenic Hu128/21 HD mouse model.^18^ Based on this study, we selected a miHTT construct that showed the strongest efficacy *in vitro* and induces a potent total HTT knock-down in the striatum and cortex of the Hu128/21 HD mice.^18^ For the allele-selective HTT knock-down, we selected the miSNP50T and miSNP67T constructs that showed strongest efficacy *in vitro*.

Although several transgenic or knock-in HD animal models have been established and used for preclinical testing, none completely recapitulates the neuropathology that occurs in HD patients.^19, 20, 21^ Therefore, a combination of several *in vivo* preclinical studies is required to address the necessary treatment outcome and safety measures for HD before entering the clinic. In respect to the latter, the present study was designed to evaluate the on-target efficacy of continuously expressed miHTT, miSNP50T, and miSNP67T constructs in suppressing the neuropathology associated with HD using an acute lentiviral (LV) HD rat model. To generate the HD rat model, wild-type rats were injected intrastriatally with a LV expressing a chimeric mutant HTT fragment, which is shown to induce local formation of mutant HTT aggregates followed by severe neuronal dysfunction at two months post-infection.^22^ Therefore, this model allowed us to address the HD treatment response downstream of the mutant HTT protein in a larger rodent brain. Moreover, the chimeric LV sequence enabled us to study both total and allele-selective approaches in the context of mechanistic efficacy and gene specificity.^23^

## Results

### miHTT-155 delivered by an AAV5 vector suppressed mutant HTT aggregate formation and DARPP-32-associated neuronal dysfunction in HD rats

**Figure 1:**
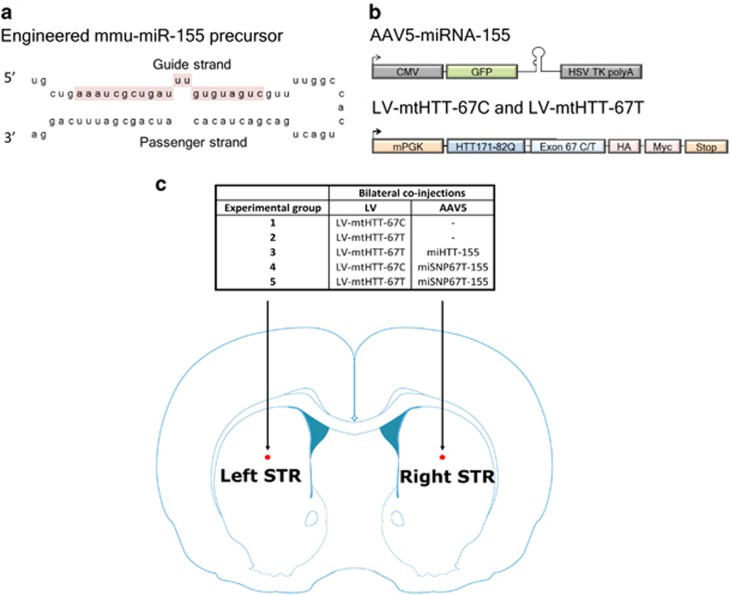
Design of a proof-of-concept study using the AAV5-miHTT-155 and AAV5-miSNP67T-155 vectors in an acute HD rat model. (**a**) The structure and sequence of the engineered mmu-pre-miR-155 precursor used in the study with the highlighted guide strand in pink. (**b**) Schematic representation of the AAV5-miHTT-155 and AAV5-miSNP67T-155 expression cassettes; and LV-mtHTT-67C and LV-mtHTT-67T expressing the chimeric mutant HTT sequences. (**c**) Bilateral co-injections in the striatum (STR) of rats with LV-mtHTT-67C or LV-mtHTT-67T, and AAV5-miHTT-155 or AAV5-miSNP67T-155 vectors. The experimental groups and injection sites are outlined.

To assess distribution, mechanistic efficacy and allele selectivity of the therapeutic miRNA sequences, we initiated a pilot study in the acute LV HD rat model using the miHTT construct for the total HTT knock-down and the miSNP67T construct for the allele-selective knock-down. Both miHTT and miSNP67T constructs were previously designed in the engineered mmu-miR-155 precursor, named miHTT-155 and miSNP67T-155 (Figure 1a). The miHTT and miSNP67T expression cassettes also contained a sequence encoding green fluorescent protein (GFP) to assess transduction efficiency (Figure 1b).^18^

We generated AAV5 viruses carrying the miHTT and miSNP67T expression cassettes and co-injected each virus bilaterally in the striatum of rats with a LV vector encoding a chimeric human mutant HTT sequence (Figures 1b and c). The LV vector consisted of an 82-long glutamine (82Q) chain fused with the target regions for the miHTT and miSNP67T constructs, named LV-mtHTT-67T.^23^ To address the allele-selective potential of the miSNP67T construct, we included in this study a second LV vector named LV-mtHTT-67C. The AAV5-miSNP67T-155 expression product perfectly matches LV-mtHTT-67T transcripts and has one nucleotide mismatch with LV-mtHTT-67C transcripts at the single nucleotide polymorphism (SNP) rs362307. Therefore, this system enabled us to test the allele selectivity based on only a single SNP. The mutant HTT protein that is expressed from both LV vectors is known to cause HD-like neuronal dysfunction.^22^

**Figure 2:**
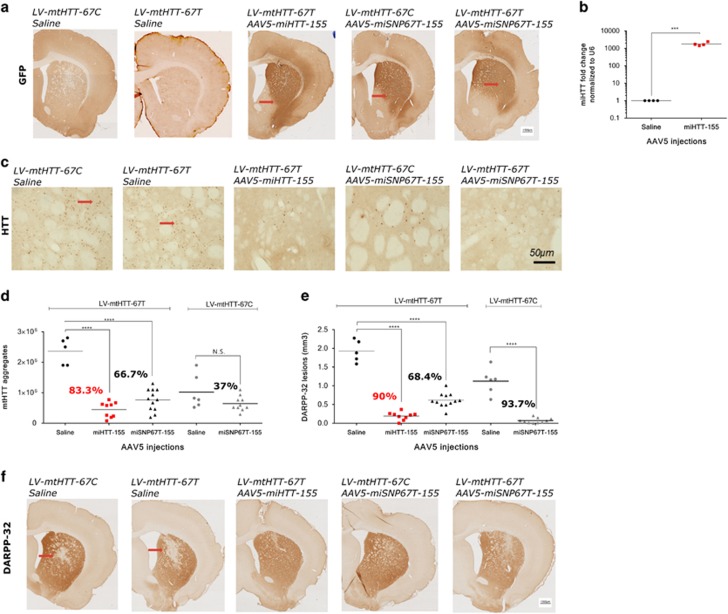
Phenotypic improvement of HD neuropathology following AAV5-miHTT-155 and AAV5-miSNP67T-155 injections in HD rats. (**a**) IHC with an anti-GFP antibody showing AAV5 distribution. A representative picture of the right hemisphere is shown and GFP-positive areas are depicted by a red arrow 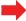. (**b**) miHTT-specific TaqMan assay to determine miHTT fold change in the striatum of AAV5-miHTT-155 injected rats compared with the saline-treated rats (*n*=4). miHTT values are presented as the distribution plot with the mean of the values following normalization to U6 levels. (**c**) IHC with an anti-HTT antibody showing the mutant HTT aggregates. A representative picture of the right hemisphere is shown and mutant HTT aggregates are represented by a red arrow 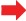. (**d**) Quantification of anti-HTT staining shows a reduction of mutant HTT aggregates induced by AAV5-miHTT-155 and AAV5-miSNP67T-155 vectors in the striatum (*n*=5-12). The reduction (%) of mutant HTT aggregates is relative to the saline control. (**e**) Quantification of DARPP-32 staining shows a reduction in neuronal dysfunction induced by AAV5-miHTT-155 and AAV5-miSNP67T-155 vectors in the striatum (*n*=5-12). The reduction (%) in neuronal dysfunction is relative to the saline control. (**f**) IHC against DARPP-32 showing neuronal dysfunction. A representative picture of the right hemisphere is shown and DARPP-32-negative areas are depicted by a red arrow 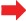. All data were analyzed using one-way ANOVA. NS, non-significant, *P*>0.05; *P⩽0.05; **P⩽0.01; ***P⩽0.001; ****P⩽0.0001. The values were calculated as a mean±s.d.

Two months post-injection, rats were euthanized and the brain tissues were analyzed for AAV5 vector distribution, mature miHTT expression, on-target silencing efficiency measured by mutant HTT aggregate formation and DARPP-32-associated neuronal dysfunction. To evaluate AAV5 vector distribution in the HD rat brain, we performed immunohistochemistry (IHC) against GFP on the fixed striato-cortical sections (Figure 2a). For all AAV5 constructs, we observed broad striatal with partial cortical GFP distribution. Consistent with the latter, we observed ~1800 times more miHTT molecules in the AAV5-miHTT-155-injected rats compared with the saline control (Figure 2b).

The accumulation of mutant HTT aggregates in the brain is a hallmark of the HD neuropathological process.^7^ To validate on-target silencing efficiency of the AAV5-miHTT-155 and AAV5-miSNP67T-155 expression products, we performed IHC using an anti-HTT antibody that binds to the mutant HTT (Figure 2c). We observed significantly fewer mutant HTT aggregates in the HD rat striata injected with the AAV5-miHTT-155 (0.4 × 10^6^±0.2 × 10^6^, *P*⩽0.0001) or AAV5-miSNP67T-155 viruses (0.8 × 10^6^±0.3 × 10^6^, *P*⩽0.0001) compared with the saline control (2.4 × 10^6^±0.4 × 10^6^) (Figure 2d). The AAV5-miSNP67T-155 showed no allele-selectivity as similarly low counts of mutant HTT aggregates were observed in both LV-mtHTT-67C- (0.6 × 10^6^±0.2 × 10^6^) and LV-mtHTT-67T-injected rats compared with the corresponding saline controls.

To address the effect of AAV5-miHTT-155 and AAV5-miSNP67T-155 treatments on HD-linked neuronal dysfunction, we stained for dopamine- and cyclic-AMP-regulated phosphoprotein of 32 kDa(DARPP-32), a phosphoprotein widely expressed in medium spiny neurons.^24^ Consistent with the low accumulation of mutant HTT aggregates, we observed a significant reduction of DARPP-32 lesion size two months after AAV5-miHTT-155 (0.19 mm^3^±0.11, *P*⩽0.0001) or AAV5-miSNP67T-155 (0.62 mm^3^ ± 0.19, *P*⩽0.0001) treatments compared with the saline control (1.92 mm^3^±0.29) (Figures 2e and f). AAV5-miHTT-155 was again shown to be the most effective. Moreover, consistent with the mutant HTT aggregate counts, AAV5-miSNP67T-155 showed no allele selectivity as demonstrated by a similar DARPP-32 lesion size in both LV-mtHTT-67C- (0.07 mm^3^±0.07) and LV-mtHTT-67T-injected rats relative to the saline controls. These experiments enabled us to identify the miHTT guide sequence effectively targeting HTT, which resulted in a strong suppression of mutant HTT aggregate formation and neuronal dysfunction at two months post-injection.

### Optimized miHTT-451 and miSNP50T-451 expressed from AAV5 vectors induced the mutant HTT mRNA knock-down in HD rats

**Figure 3:**
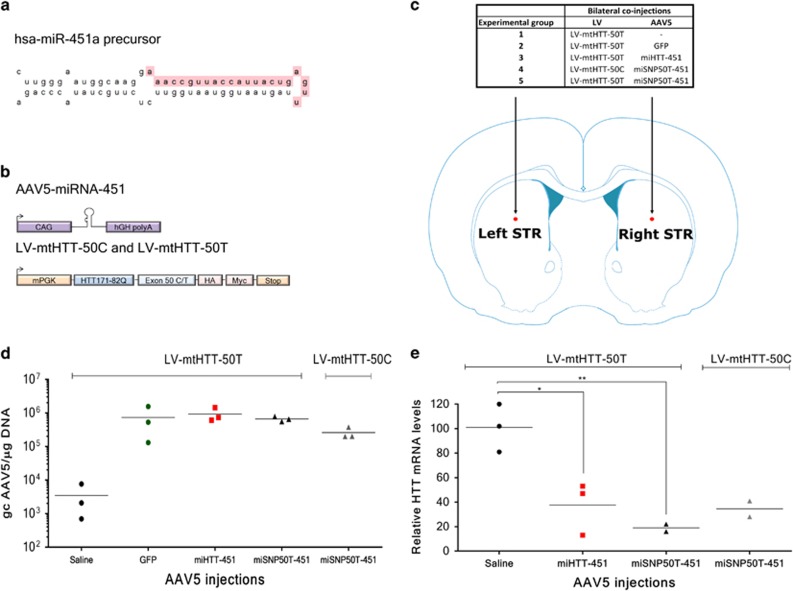
HTT mRNA knock-down induced by AAV5-miHTT-451 and AAV5-miSNP50T-451 vectors in the striatum of HD rats. (**a**) The structure and sequence of the hsa-pre-miR-451a precursor used in this study with the miRBase-predicted guide strand highlighted in pink (www.mirbase.org). (**b**) Schematic representation of the AAV5-miHTT-451 and AAV5-miSNP50T-451 expression cassettes; and LV-mtHTT-50C and LV-mtHTT-50T encoding a chimeric mutant HTT sequence with either C or T isoform of SNP rs362331. (**c**) Bilateral co-injections in the striatum (STR) of rats with LV-mtHTT-50C or LV-mtHTT-50T, and AAV5-miHTT-451 or AAV5-miSNP50T-451 vectors. The experimental groups and injection sites are outlined. (**d**) qPCR to determine AAV5 genome copies (gc) in the striatum of AAV5-miHTT-451 and AAV5-miSNP50T-451 injected rats (*n*=3), two months post-injection. Primers directed to the CAG promoter were used and the gc values were calculated based on the standard curve and considering the background signal from the negative control. (**e**) TaqMan qPCR assay shows HTT mRNA knock-down in the striatum (*n*=2-3) induced by the AAV5-miHTT-451 and AAV5-miSNP50T-451 expression products. Human HTT-specific exon-spanning primers were used and HTT values were subsequently normalized to GAPDH, an internal control set at 100%. All data were analyzed using one-way ANOVA. *P⩽0.05; **P⩽0.01; ***P⩽0.001; ****P⩽0.0001. The values were calculated as a mean±s.d.

Although the engineered mmu-miHTT-155 constructs generates the favorable miHTT effector sequence and is suitable for pilot experiments addressing efficacy and distribution, it is not optimal for clinical studies in humans due to a high risk of immune response to GFP and possibility of off-target silencing caused by the passenger strand.^18^ Previously, we showed that the silencing efficacy and processing of the miHTT construct are influenced by the pre-miHTT precursor.^18^ We identified the miHTT-451 precursor that showed no passenger strand *in vivo* opposite to the miHTT-101 and miHTT-135 precursors (Figure 3a). Moreover, the miHTT-451 construct showed efficacious HTT knock-down and no signs of toxicity. Therefore, in this study we included the miHTT-451 precursor for the total HTT knock-down. For the allele-selective approach, we optimized the miSNP50T construct designed to preferentially bind to HTT messenger RNA (mRNA) carrying the U isoform of SNP rs362331, which is associated at high frequencies with HD.^25^ This miSNP50T construct, which exhibits stronger efficacy than miSNP67T *in vitro*, was not available when the pilot study was initiated and therefore, it was introduced at this stage. Moreover, for both constructs the cytomegalovirus promoter together with GFP was replaced by the strong cytomegalovirus immediate-early enhancer fused to chicken β-actin (CAG) promoter, which has been shown to be effective in the brain.^26^ The resultant constructs are named miHTT-451 and miSNP50T-451 (Figure 3b).

To further establish mechanistic efficiency, safety, as well as allele selectivity *in vivo*, we generated AAV5-miHTT-451 and AAV5-miSNP50T-451 vectors and co-injected them bilaterally in the striatum of rats with a LV vector encoding a chimeric mutant HTT sequence with an 82Q chain and carrying target regions for the miHTT and miSNP50T constructs, named LV-mtHTT-50T (Figures 3b and c). In order to address the allele-selective potential of the miSNP50T construct, we included a second LV vector, named LV-mtHTT-50C. The AAV5-miSNP50T-451 expression product was designed to have one nucleotide mismatch with the LV-mtHTT-50T transcripts and two nucleotide mismatches with the LV-mtHTT-50C transcripts. Therefore, this system enabled us to test the allele selectivity based on only a single SNP. As negative controls, we included a GFP expressed from the CAG promoter and saline control. Two months post-injection, rats were euthanized and the brains were processed to assess on-target efficacy, HD-like neuronal dysfunction, and immune reaction.

To measure AAV5 vector DNA in striata, we performed real-time quantitative PCR (qPCR) with primers directed towards the CAG promoter (Figure 3d). We observed high and comparable vector DNA in the striatal homogenates from AAV5-GFP-, AAV5-miHTT-451- and AAV5-miSNP50T-451-injected rats. Similar to TaqMan assays (data not shown), we detected a background level of ~3000 genome copies in the saline control, which was considered for calculations of final vector DNA. Furthermore, the latter inversely correlated with the human-specific HTT mRNA knock-down from the same tissue homogenate demonstrated by TaqMan RT-qPCR (Figure 3e). We detected 62.3%±21.6 HTT mRNA knock-down in AAV5-miHTT-451- and 81%±4.3 HTT mRNA knock-down in AAV5-miSNP50T-451-injected rats as compared with the saline control. We observed similar HTT mRNA knock-down by AAV5-miSNP50T-451 in LV-mtHTT-50T-injected rats compared with LV-mtHTT-50C-injected rats where 65.5%±9.2 HTT mRNA reduction was detected.

### On-target silencing efficiency of AAV5-miHTT-451 and AAV5-miSNP50T-451 correlates with suppression of DARPP-32-associated neuronal dysfunction in HD rats

**Figure 4:**
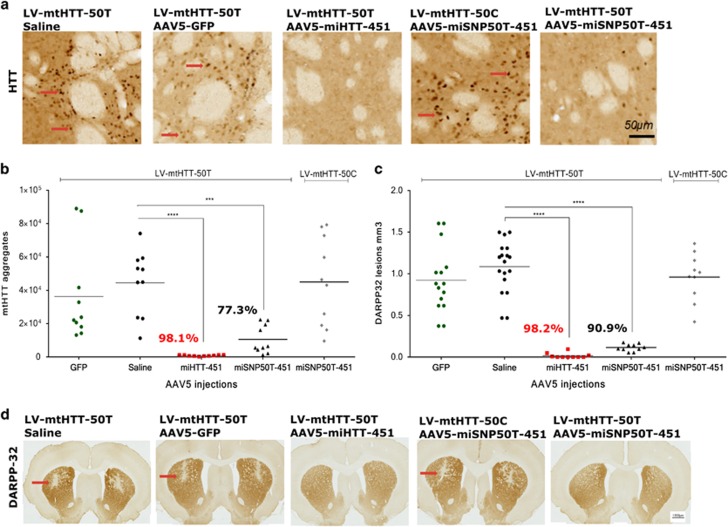
Phenotypic improvement of HD neuropathology following AAV5-miHTT-451 and AAV5-miSNP50T-451 injections in HD rats. (**a**) IHC using an anti-HTT antibody showing the mutant HTT aggregates. A representative picture of both hemispheres is shown and the mutant HTT aggregates are depicted by a red arrow 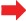. (**b**) Quantification of anti-HTT staining shows a reduction of mutant HTT aggregates induced by the AAV5-miHTT-451 and AAV5-miSNP50T-451 treatment in the striatum (*n*=10). The reduction (%) of mutant HTT aggregates is relative to the saline control. (**c**) Quantification of DARPP-32 staining shows reduced neuronal dysfunction following AAV5-miHTT-451 and AAV5-miSNP50T-451 injections in the striatum (*n*=10–18). The reduction (%) in neuronal dysfunction is relative to the saline control. (**d**) IHC against DARPP-32 showing neuronal dysfunction. A representative picture of both hemispheres is shown and DARPP-32-negative areas are depicted by a red arrow 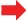. All data were analyzed using one-way ANOVA. *P⩽0.05; **P⩽0.01; ***P⩽0.001; ****P⩽0.0001. The values were calculated as a mean±s.d.

To establish the ability of our optimized AAV5-miHTT-451 and AAV5-miSNP50T-451 vectors to suppress formation of mutant HTT aggregates, we analyzed striato-cortical sections by IHC using an anti-HTT antibody (Figure 4a). Here, in the AAV5-miHTT-451-injected rats almost no detectable mutant HTT aggregates were observed (843±359, *P*⩽0.0001), whereas the aggregates were abundant in the GFP (3.6x10^4^±2.9x10^4^) and saline (4.4x10^4^±2x10^4^) controls (Figure 4b). Based on the number of mutant HTT aggregates, AAV5-miSNP50T-451 showed stronger silencing of the matched LV-mtHTT-50T expression product (1x10^4^±0.8x10^4^, *P*⩽0.001) compared with the mismatched LV-mtHTT-50C (4.5x10^4^±2.6x10^4^), suggesting allele-selective potential.

During development of symptomatic HD, the appearance of mutant HTT aggregates precedes the death of medium spiny neurons located in the striatum.^22^ Therefore, we addressed the potential of our AAV5-miHTT-451 and AAV5-miSNP50T-451 vectors to suppress DARPP-32-associated neuronal dysfunction by performing IHC against DARPP-32 (Figures 4c and d). Consistent with lowering of mutant HTT aggregates, AAV5-miHTT-451 (0.02 mm^3^±0.03, *P*⩽0.0001) and AAV5-miSNP50T-451 (0.12 mm^3^±0.04, *P*⩽0.0001) treatments significantly suppressed the partial striatal lesion induced by the LV-mtHTT-SNP50T expression product compared with the GFP (0.92 mm^3^±0.39) and saline controls (1.09 mm^3^±0.31). In contrast, AAV5-miSNP50T-451 treatment did not show strong reduction in the size of DARPP-32 lesions in LV-mtHTT-SNP50C-injected rats (0.96 mm^3^±0.29), demonstrating allele selectivity.

### AAV5 viruses delivering the miHTT-451 or miSNP50T-451 precursors did not induce an overt immune response via GFAP and Iba1 activation

**Figure 5:**
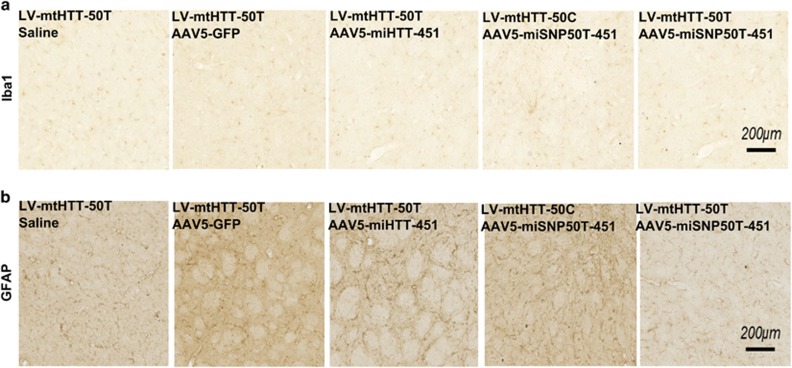
No apparent activation of microglia and astrocytes following AAV5-miHTT-451 and AAV5-miSNP50T-451 injections in HD rats. (**a**) IHC against Iba1 to show microglial activity. A representative picture of the right hemisphere is shown. (**b**) IHC against GFAP to show the astrocyte activity. A representative picture of the right hemisphere is shown.

Although AAV-based gene therapy is an attractive approach for the delivery of gene products, such as therapeutic miRNAs, and overall has demonstrated low toxicity to date, activation of the immune response should be addressed for each therapeutic candidate in a given target tissue. To evaluate the immune response to our AAV5 vectors expressing therapeutic miRNA precursors in the brain, we analyzed the activation of microglia and infiltration of astrocytes in the injected areas by IHC using an anti-ionized calcium-binding adapter molecule 1 (Iba1) and anti-glial fibrillary acidic protein (GFAP) antibody, respectively (Figures 5a and b). We found similar levels of immunoreactivity in AAV5-miHTT-451- and AAV5-miSNP50T-451-treated rats compared to the saline control, indicating no apparent activation of immune reaction via microglia and astrocytes.

### AAV5-miHTT-451 and miSNP50T-451 constructs do not generate a passenger strand *in vivo*

**Figure 6:**
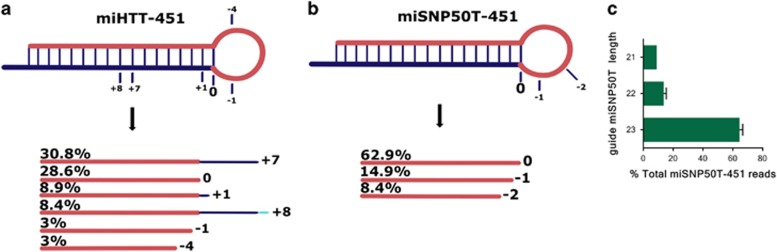
Next-generation sequencing analysis of the pre-miHTT-451 and pre-miSNP50T-451 processing patterns shows no passenger strand *in vivo*. (**a**) Sequence distribution (%) of reads mapping to pre-miHTT-451 *in vivo*.^18^ The predicted guide strand is indicated in red and a mismatch with the reference sequence in light blue. (**b**) Sequence distribution (%) of reads mapping to pre-miSNP50T *in vivo*. The predicted guide strand is indicated in red and a mismatch with the reference sequence in light blue. (**c**) The length distribution of reads mapping to the pre-miSNP50T precursor (*n*=2). For the read alignments, up to 3 mismatches with the reference sequence were allowed. Reads represented with less than 3% were excluded from the figure.

Previously, we showed that the miHTT-451 construct does not generate a passenger strand and is not toxic probably due to higher specificity *in vivo* (Figure 6a).^18^ The absence of the passenger strand from the miR-451 scaffold provides a better safety profile for therapeutic shRNAs or miRNAs since it reduces the chance for off-target suppression of other genes by the passenger strand.^18, 27, 28^ To establish the absence of a passenger strand for the miSNP50T-451 construct *in vivo*, we analyzed the processing patterns in the striatum of AAV5-miSNP50T-451-injected rats by next-generation sequencing. We observed consistent processing patterns between the analyzed samples (Figure 6b). The most abundant reads were 23 nucleotides long and belonged to the miSNP50T guide strand (Figure 6c). We did not observe any reads belonging to the passenger strand, confirming the absence of a passenger strand associated with the miR-451 precursor.

Based on this study, we identify the AAV5-miHTT-451 construct as the most efficient candidate for the gene therapy approach targeting HTT by showing a robust suppression of crucial HD pathological features in the rat model of HD.

## Discussion

In the current study, we have demonstrated the feasibility of total and allele-selective HTT silencing induced by therapeutic miRNAs delivered to the brain. Intracerebral administration of AAV5 vectors delivering the miHTT candidates for total HTT knock-down or the miSNP50T and miSNP67T candidates for the allele-selective approach resulted in suppression of mutant HTT aggregates and prevention of DARPP-32-associated neuronal dysfunction in rat striata. The AAV5-miHTT-451 construct recapitulated the *in vitro* observations by demonstrating the strongest efficacy *in vivo*.

The LV HD rat model very closely and rapidly reproduces the critical pathogenic features of HD: the mutant HTT aggregation and striatal neuronal dysfunction. Although this model does not permit study of improvement in HD-like behavioral symptoms, it provides a means to preselect therapeutic candidates to continue with high-cost preclinical studies in large animals that carry on for several months to years. Previous studies with shRNAs showed equivalent silencing efficacy of the chimeric HTT constructs in rats and HEK-293T cells compared with silencing of the endogenous full-length human HTT in HD-derived neuronal cultures, suggesting that accessibility of silencing agents to the chimeric HTT constructs resembles the natural situation.^23^ Moreover, the rapid severity of DARPP-32-associated neuronal dysfunction challenges the read-out efficacy; but on the other hand, it strengthens confidence in case efficacy is demonstrated.

Most HD patients are heterozygous at the HTT locus and therefore many studies attempt to target only the mutant HTT. However, the nature of the HD mutation makes allele-selective HTT silencing challenging. To distinguish between the mutant and healthy HTT alleles, most of the therapeutic interventions so far have focused on targeting the heterozygous HD-associated SNPs or CAG expansions.^23, 25, 29, 30, 31, 32, 33^ However, not all HD patients have the identified common SNP isoforms linked to HD. Therefore, more than one product would be needed to fully address the HD patient population. For instance, one study showed that five therapeutic products targeting the isoforms of three different SNPs are required to treat three-quarters of the United States and European HD patient populations.^31^ Moreover, as a personalized medicine, additional genotyping will be required to identify suitable HD candidates for a given clinical trial. Furthermore, the allele selectivity based on targeting the CAG expansions depends on a difference between the consecutive CAGs located on the mutant and healthy HTT alleles. Nevertheless, it has not shown to be more efficacious than the SNP approach, when simultaneously tested.^18, 32^ Ultimately, the presence of CAG repeats in more than 66 genes increases the chance for off-targeting, making it a less attractive target for clinical testing.^34^ Consequently, the allele-selective approach still faces obstacles to advance into the clinical development.

In this study, we identified AAV5-miSNP50T-451 that showed allele-selective silencing of the HTT allele carrying the T isoform of SNP rs362331. The ongoing experiments with the Hu128/21 mice that express full-length human HTT with the U isoform of SNP rs362331 will provide more insights into efficacy and allele-selectivity of the AAV5-miSNP50T-451. The AAV5-miSNP67T-155 construct showed no allele-selectivity *in vivo*. mfold prediction analysis indicated that the secondary RNA structures of miHTT, miSNP67T and miSNP50T target sites do not form stable duplexes (data not shown), supporting their similar accessibility to the therapeutics. Previously, it has been reported that allele-selective potential of artificial miRNAs observed *in vitro* may not be retained *in vivo*.^35^ In general, miRNA–mRNA interactions are determined by the sequence composition that influences the thermodynamics, such as the melting temperature, of the duplex RNA.^36^ miSNP67T binds to 52%-rich GC region, which could enhance the miRNA:mRNA pairing stability and ultimately contribute to no allele-selectivity based on a single SNP observed *in vivo*.

Most HTT lowering strategies intend to suppress both HTT alleles and the first ongoing clinical trial with antisense oligonucleotides employs this approach (https://clinicaltrials.gov/). Preclinical studies using HD animal models and non human primates indicate that there is a therapeutic window where total HTT knock-down is safe and yet beneficial for HD patients.^37, 38, 39^ Importantly, this strategy would provide a treatment for all HD patients independent from their genotype. In this study, we showed that the miHTT construct processed from the miR-451 scaffold is able to strongly suppress mutant HTT aggregation and ultimately dramatically reduce the generation of striatal lesions at two months post-injection. Moreover, we did not observe immune reaction to the AAV5-miHTT-451 vector via astrocytes or microglia activation, suggesting a favorable safety profile.

Altogether, these data provide strong evidence for the therapeutic efficacy of the AAV5-miHTT-451 vector by inducing functional improvements downstream in the HD pathological process without raising safety concerns in a rodent model of HD. These promising results support our ongoing efforts for the development of an HD-modifying treatment by silencing the human HTT gene.

## Materials and methods

### HTT target sequences

Homo sapiens HTT mRNA, complete CDS (gb|L12392.1|HUMHDA) obtained from http://blast.ncbi.nlm.nih.gov/Blast.cgi, was used to identify target sequences for the artificial miHTT targeting exon 1, the miSNP50T construct binding to the U isoform of SNP rs362331, and the miSNP67T construct targeting the U isoform of SNP rs362307.

### DNA constructs

To generate the miHTT-155 and miSNP67T-155 constructs, their guide sequences targeting HTT transcripts were embedded into the engineered murine pre-miR-155 backbone of pcDNA6.2-GW/EmGFP-miR (Invitrogen, Carlsbad, CA, USA) by annealing complementary oligonucleotides (Qiagen, Valencia, CA, USA) followed by ligation into the linearized pcDNA6.2 plasmid.^18^

To generate miHTT-451 and miSNP50T-451 constructs, the guide and passenger strand sequences were incorporated in the hsa-pri-miR-451a scaffold. 200 nucleotides long 5′ and 3′ encompassing flanking regions were included with EcoRV and BamHI restriction sites and the complete sequences were ordered from GeneArt gene synthesis (Invitrogen). In these constructs, the cytomegalovirus promoter was replaced by the CAG promoter (Inovio, Plymouth Meeting, PA).^18^

### AAV5 vector production

AAV5 vectors expressing miHTT, miSNP50T, miSNP67T and GFP were produced by baculovirus-based AAV production system (uniQure, Amsterdam, The Netherlands). The artificial miRNA cassettes were cloned in a transfer plasmid in order to generate an entry plasmid. The expression cassettes were inserted in a recombinant baculovirus vector by homologous recombination and clones were selected by plague purification. The recombinant baculovirus containing the cassettes were further amplified till the passage 6 and clones screened for the best production and stability by PCR and qPCR. To generate AAV5, infections with different recombinant baculoviruses containing the vector genome, the replicon enzyme and the capsid protein were performed. The cells were lysed 72 h after infection and the crude lysate was treated with Benzonase (50 U ml^−1^; Merck, Darmstadt, Germany) for 1 h at 37 °C. AAV5 was purified on AVB Sepharose column (GE Healthcare, Little Chalfont, UK) using AKTA Explorer purification system (GE Healthcare) and the genome copy titer was determined by qPCR.

### Lentiviral vector production

LVs were produced in HEK-293T cells with the four-plasmid system, as previously described.^40^ Human immunodeficiency virus type-1 vectors were pseudotyped with the vesicular stomatitis virus glycoprotein envelope, concentrated by ultracentrifugation and resuspended in phosphate-buffered saline (PBS, Gibco, Life Technologies, Zug, Switzerland) supplemented with 1% bovine serum albumin (BSA, Sigma-Aldrcich, Buchs, Switzerland). The viral particle content of each batch was determined by p24 antigen enzyme-linked immunosorbent assay (p24 ELISA, RETROtek, Kampenhout, Belgium). Viral stocks were stored at −80 °C. Lentiviral vectors encoding the mutant HTT fragments were used at 300 ng of p24. Based on transcriptional start site from the PGK promoter and the presence of polyA+ signal in the 3′LTR of the lentiviral vector, the mRNAs expressed from the LV-mtHTT-50T and LV-mtHTT-67 constructs are expected to be ~1993 bp and 2059 bp, respectively.^41^

### Animals

Adult male Sprague Dawley rats (200 g; OFA, Charles River, Oncins, France) were housed in a temperature-controlled room and maintained on a 12 h day/night cycle. Food and water were available *ad libitum*. All experiments were carried out in accordance with the European Community directive (86/609/EEC) for the care and use of laboratory animals as well as the Swiss animal welfare laws under the authorization no. VD 2487 and 2889 from the Service de la consommation et des affaires vétérinaires du Canton de Vaud, Switzerland.

### Stereotaxic injections

The animals were anesthetized using a solution containing 75 mg kg^−1^ ketamine (Ketasol, Graeub, Bern, Switzerland) and 10 mg kg^−1^ xylazine (Rompun, Bayer Health Care, Uznach, Switzerland) administered i.p. The rat was placed in the stereotaxic frame (model 963 Ultra Precise Small Animal Stereotaxic Instrument, David Kopf Instruments, Tujunga, CA, USA). Viral vectors were stereotaxically injected into the striatum of animals through a 34-gauge blunt-tip needle (Phymep, Paris, France) linked to a Hamilton syringe (Hamilton Medical AG, Bonaduz, Switzerland) by a polyethylene catheter. The stereotaxic coordinates for an intrastriatal injection were: 0.5 mm rostral to bregma, 3 mm lateral to midline and 5 mm from the skull surface. For the infections, 300 ng p24 antigen of lentiviral vectors were mixed with 6.5 × 10E^10^ gc of AAV5 in 4 μl and injected at 0.2 μl min^−1^. The injections were not performed in a blind manner. The rats received 2 mg ml^−1^ acetaminophen (Dafalgan, UPSA, Agen, France) in water for the 3 days following the intervention.

### Histology

The rats were euthanized by an overdose of sodium pentobarbital 150 mg kg^−1^ (B-Braun Mediacal SA, Sempach, Switzerland) and transcardially perfused at a rate of 20 ml min^−1^ with 100 ml of 1x PBS and then with 300 ml of 4% PFA. Brains were removed and post-fixed by incubation in 4% PFA for 24 h at 4 °C and cryoprotected by incubation in 20% sucrose (Sigma-Aldrich, Buchs, Switzerland) in 1x PBS for 24 h and in 30% sucrose in 1x PBS for 24 h. Brains were frozen on dry ice and then stored at −80 °C. For RNA analysis, the animals were euthanized and the brains were snap-frozen in liquid nitrogen.

25 μm-thick coronal brain sections were cut on a sledge microtome with a freezing stage at −30 °C (Leica SM2010R, Biosystems Switzerland, Nunningen, Switzerland). Sections throughout the striatum were collected and kept in tubes, as free-floating sections in anti-freeze solution (25% glycerol (Sigma-Aldrich, Buchs, Switzerland), 30% Ethylene glycol (Merck, Nottingham, UK), 25% 1x PBS and 20% nanopure water).

### Immunostaining

Free-floating sections were washed three times, for 10 min each, in Tris-buffered saline (TBS, 10 mM Tris pH=7.6 and 0.9% NaCl Sigma-Aldrich, Buchs, Switzerland). Non-specific peroxidases were inactivated by incubation in quenching solution (TBS supplemented with 3% H_2_O_2_ and 10% methanol, Sigma-Aldrich, Buchs, Switzerland) for 15 min at room temperature. Sections were washed twice, for 10 min each, in TBS, and then for 10 min in TBS-0.1% Triton X-100 (TBST). Sections were blocked by incubation in Tris-high salt-buffer (THST, 50 mM Tris pH=7.6, 0.5 M Nacl, 0.5% Triton X-100,) for anti-HTT antibody or TBST-5% NGS for DARPP-32 antibody during 1 h at room temperature and were then incubated overnight at 4 °C in TBST-1% BSA with antibodies. The following primary antibodies were used: Goat polyclonal anti-HTT antibody (1/1000, Sicgen, Coimbra, Portugal), rabbit polyclonal anti-DARPP-32 antibody (1/1000, Cell Signaling Technology Europe, Leiden, The Netherlands), rabbit polyclonal anti-GFP antibody (1/100 000, Abcam, Cambridge, UK), rabbit polyclonal anti-IbaI antibody (1/25 000, Wako Chemicals, Richmond, USA) and rabbit polyclonal anti-GFAP antibody (1/500, Dako, Baar, Switzeland). After incubation with the primary antibody, the sections were washed three times, for 10 min each, in TBS and then incubated for 1 h at room temperature in TBST-1% BSA with secondary antibodies. The following secondary antibodies were used: biotinylated donkey anti-goat IgG (1/1000, Jackson, Pennsylvania, USA) and biotinylated goat anti-rabbit IgG (1/400, Vector Lab Inc, Burlingame, USA). Sections were washed three times, for 10 min each, in TBST and incubated for 1 h at RT in ABC (Vector Lab Inc). Samples were washed twice, for 10 min each, in TBS and were then incubated with nickel chloride and diaminobenzidine (DAB, Vector Lab Inc) for antibody detection. Sections were mounted on Superfrost Ultraplus slides. Sections were allowed to dry in a laminar flow hood and were dehydrated (70% ethanol in nanopure water, 95% ethanol in nanopure water, 100% ethanol, 100% ethanol and xylene) before mounting in Eukitt (Kindler GmbH, Freiburg, Germany).

### Image acquisition and quantification

Images were obtained with a digital camera (3CCD Hitachi HV-F202SCL) on a slide scanner microscope (× 20 objective, Zeiss axioscan Z1). Measurements were made with Image J software (http://rsb.info.nih.gov/ij/, NIH, Bethesda, USA) for aggregates and Zeiss Zen Lite 2 (blue Edition; Carl Zeiss AG, Germany) for DARPP32 lesion. An Image J macro was developed to count the number of aggregates. The aggregates were identified on the basis of their intensity relative to the background, their size and their circularity. The same intensity, size and circularity parameters were used for all samples.

### Quantification of mtHTT aggregates and DARPP-32 lesions

The number of mutant HTT aggregates in the striatum was automatically counted in 1 in 8 sections with analyze particle plugin on ImageJ (Supplementary Material and Method 1). Cut-off with minimum size and maximum pixel area size was applied to exclude non-aggregates in the ROI. The total number of aggregates was estimated by multiplying these densities by 8. The loss of DARPP-32 expression was analyzed by collecting digitized images sections (1 in 8 sections separated by 200μm). Lesion areas in each section were determined as regions poor in DARPP-32 staining relative to the surrounding tissue. The lesion volume for each animal was quantified with the following formulae: lesion in mm^3^ = (mean size in μm^2^ × number of counted section × inter-section distance)/1 × 10E9. Means and s.d.’s were calculated for each group. The quantifications were performed in a blind manner.

### Quantitative real-time PCR

Rat striata were crushed using CryoPrep System (Covaris, Woburn, MA, USA) and the powder was divided for RNA and DNA analyses. For RNA isolation, the striatal powder was homogenized in Trizol using gentleMACS Dissociator (Miltenyi Biotec) and total RNA was isolated from Trizol according to the manufacturer’s protocol (Invitrogen). To remove genomic DNA, RNA was treated with dsDNase (Thermo Scientific, Waltham, MA, USA). Quantitative real-time PCR reaction was performed to detect HTT mRNA knock-down using TaqMan gene expression assays (Applied Biosystems, Foster City, CA, USA), human HTT (Hs00918134_m1), rat GAPDH (Rn01462662_g1); and miHTT expression levels using custom TaqMan microRNA expression assay or U6 snRNA (001973; Applied Biosystems). The expression level of each gene was normalized to either endogenous GAPDH or U6 snRNA levels. Fold change/percentages in HTT mRNA knock-down or miHTT expression were calculated based on 2^DDCT method (Supplementary Material and Method 2).

For the genomic DNA isolation, the striatal powder was processed using DNeasy96 Blood and Tissue kit (Qiagen). qPCR reaction was performed to detect AAV5 vector titers using a standard line and SYBR Green protocol (Applied Biosystems). Forward primer sequence: 5′-GAGCCGCAGCCATTGC-3′ and reverse primer sequence: 5′-CACAGATTTGGGACAAAGGAAGT-3′. Based on the standard line, genome copies per microgram DNA were calculated.

### Next-generation sequencing data analysis

The raw sequencing data were produced as previously described.^18^ Small RNA raw data sets were analyzed using the CLC Genomics Workbench 6 (Qiagen). All reads containing ambiguity N symbols, reads shorter than 10 nucleotides, longer than 45 nucleotides and reads represented less than 10 times were discarded. The obtained unique small RNA reads were aligned to the reference sequences of the pre-miHTT and pre-miSNP50T constructs with a max. of 3 nucleotide mismatches allowed. The percentages of reads based on the total number of reads matching the reference sequences were calculated.

### Sample sizes, calculations and statistical analysis

Sample sizes of saline *n*=18, GFP *n*=16, AAV5-miHTT-155 *n*=18, AAV5-miHTT-451 *n*=16, AAV5-miSNP67T-155 *n*=16 and AAV5-miSNP50T-451 *n*=16 represent the number of striata injected per experimental group. The sample size was chosen to consider statistical variability due to surgical procedure based on previous studies. All experiments involving animals were performed once. The only exclusion criterion was if a problem was encountered during the injection procedure. The knock-down percentages were calculated using the following formula: Knock-down (%)=[AAV5-miRNA treatment]/[saline treatment] × 100%. Data were analyzed using one-way ANOVA. *P⩽0.05; **P⩽0.01; ***P⩽0.001; ****P⩽0.0001. Differences were considered significant when *P*⩽0.05. The values were calculated as a mean±s.d.
