# Supplementary material for: AAV5-miHTT gene therapy demonstrates suppression of mutant huntingtin aggregation and neuronal dysfunction in a rat model of Huntington’s disease
Source: Gene Ther. 2017 Sep 14;24(10):630–9. doi: 10.1038/gt.2017.71 (PMC5658675; doi:10.1038/gt.2017.71)
Supplement: Supplementary Information [file gt201771x2.docx]

**ImageJ macro**

setBatchMode(true);

imgArray = newArray(nImages);

for (i=0; i<nImages; i++) {

selectImage(i+1);

imgArray[i] = getImageID();

 }

for (i=0; i< imgArray.length; i++) {

selectImage(imgArray[i]);

run("8-bit");

setAutoThreshold("MaxEntropy dark");

//run("Threshold...");

setAutoThreshold("MaxEntropy dark");

//setThreshold(0, 88);

setOption("BlackBackground", false);

run("Convert to Mask");

run("Analyze Particles...", "size=10-160 summarize add");
